# P-578. Foodborne outbreak at a public school in Mangystau region, Kazakhstan, 2024

**DOI:** 10.1093/ofid/ofaf695.792

**Published:** 2026-01-11

**Authors:** Asel Kurbanova, Dilnaz Aitbaeva, Ulyana Gubareva, Aidana Tulemagambetova, Saya Gazezova, S E V A K ALAVERDYAN, Alexander Millman, Roberta Horth, Dilyara Nabirova

**Affiliations:** Central Asia FETP, Kunayev, Almaty oblysy, Kazakhstan; Central Asia FETP, Kunayev, Almaty oblysy, Kazakhstan; Central Asia FETP, Kunayev, Almaty oblysy, Kazakhstan; Central Asia FETP, Kunayev, Almaty oblysy, Kazakhstan; Central Asia Field Epidemiology Training Program, Almaty, Almaty, Kazakhstan; American University of Armenia, Dushanbe, Yerevan, Armenia; CDC, Kazakhstan, Almaty, Almaty, Kazakhstan; US Centers for Disease Control and Prevention, Dulles, Virginia; CDC Central Asia office, Almaty, Almaty, Kazakhstan

## Abstract

**Background:**

On 7 September 2024, 141 students and staff members at a public school in Kazakhstan reported gastrointestinal illness. All had eaten daily at the school canteen. We investigated the outbreak and suggested measures to prevent similar future outbreaks.

**Table 1.**
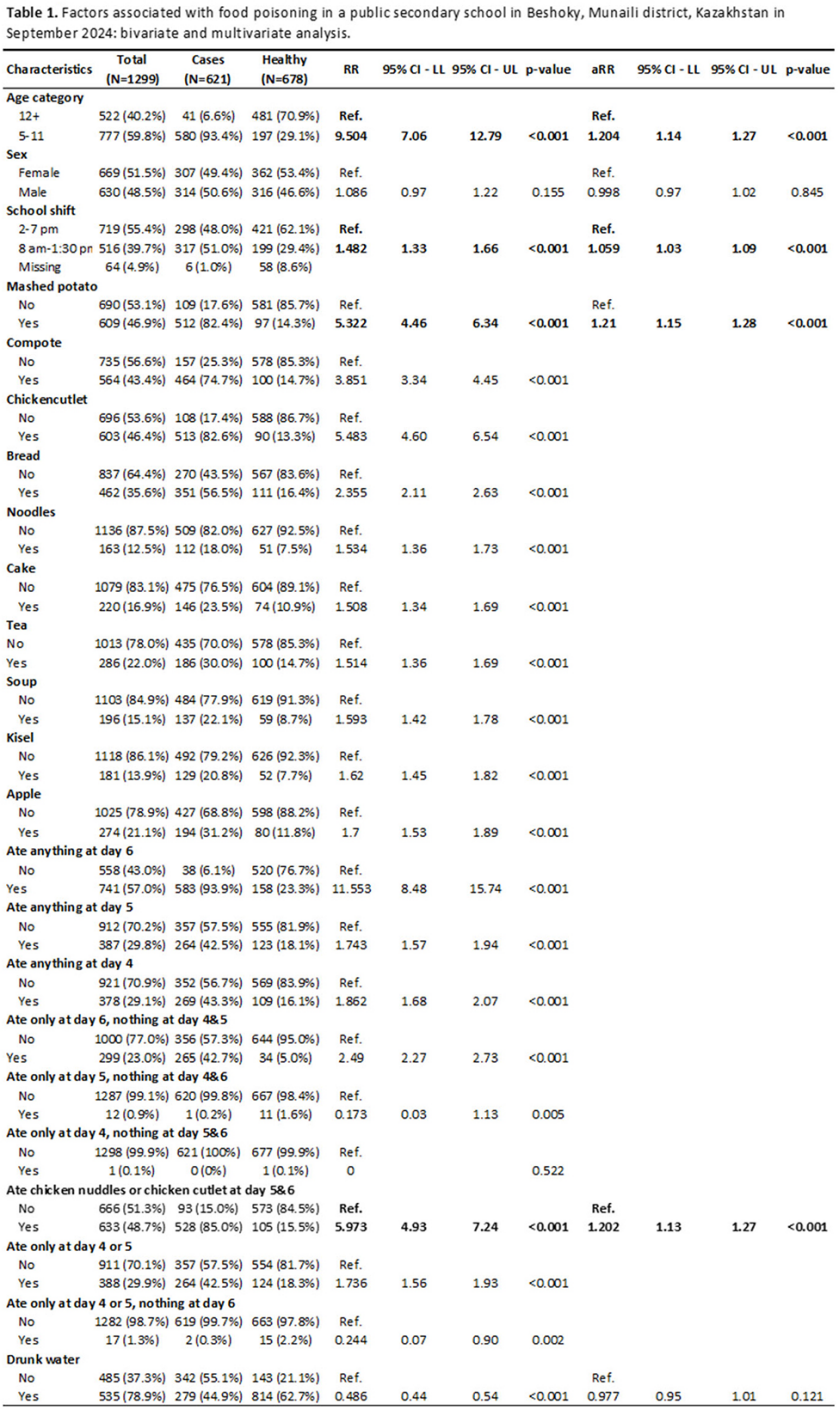
Factors associated with food poisoning in a public secondary school in Beshoky, Munaili district, Kazakhstan in September 2024: bivariate and multivariate analysis. RR: Relative risk 95% CI - LL: Confidence interval low limit 95% CI - UL: Confidence interval upper limit aRR: Adjusted relative risk P-value of Z test; all variables in the table are included in the Poisson regression model.

**Methods:**

We conducted a retrospective cohort study of students and staff who ate in the canteen during September 4-6, 2024. Cases were anyone experiencing diarrhea, vomiting, or abdominal pain from September 4–13. We interviewed consenting staff, canteen food handlers, and students, and reviewed medical records for those hospitalized. We collected participant and food handler samples [stool, rectal swab, or vomitus], food products, and surface samples. We used multivariable Poisson regression to estimate adjusted risk ratios (aRR) for exposures associated with illness.

**Results:**

Among 1,299 cohort members, 621 (48%) cases were identified; 93% were aged 5–12 years. Symptoms peaked on September 6–7 and included bloody diarrhea (74%), abdominal pain (70%), fever ≥38°C (68%), and vomiting (58%). Illness was associated with ages 5–11 vs ≥12 years (aRR=1.2, 95%CI=1.1–1.3), first-shift school attendance vs second-shift (aRR=1.1, 95%CI=1.02–1.08), consumption of mashed potatoes containing eggs (aRR=1.2, 95%CI=1.1–1.3), compote (aRR=1.1, 95%CI=1.05–1.14), and chicken served on September 5-6 (aRR=1.2, 95%CI=1.13–1.27) (Table 1). We isolated *S.sonnei, S.aureus, K.pneumoniae,* and *P.vulgaris* in 56%, 17%, 7%, and 2% of participants, respectively. Among food handler, *S.aureus* was found in 17% and *P.vulgaris* in 100%. *P.vulgaris* and *S.aureus* were detected in four chicken and mashed potato food samples from September 6. In total, 528 (85%) cases had eaten chicken, and 512 (82%) mashed potatoes. Environmental assessment revealed numerous food safety violations (cross-contamination, food storage temperatures, hygiene practices).

**Conclusion:**

The investigation highlighted critical gaps in food safety and a lack of monitoring in Kazakhstan’s schools. This led to a country-wide school canteen inspection program and food safety training for 7,471 schools.

**Disclosures:**

All Authors: No reported disclosures

